# Dropout, Attrition, Adherence, and Compliance in Mood Monitoring and Ambulatory Assessment Studies for Depression and Bipolar Disorder: Systematic Review and Meta-Analysis

**DOI:** 10.2196/83765

**Published:** 2026-01-12

**Authors:** Laurence Astill Wright, James Roe, Boliang Guo, Richard Morriss

**Affiliations:** 1 Institute of Mental Health, University of Nottingham, Jubilee Campus, University of Nottingham Innovation Park, Triumph Road, Nottingham, NG7 2TU, United Kingdom, 44 01158231294; 2 Centre for Academic Mental Health, Population Health Sciences, University of Bristol, Bristol, United Kingdom

**Keywords:** bipolar, depression, EMA, ecological momentary assessment, ambulatory assessment, mood tracking, mood monitoring, self-monitoring, adherence, attrition, dropout, drop out, compliance

## Abstract

**Background:**

Ambulatory assessment and mood monitoring are different methods that can use novel technology to deliver a more efficient, flexible, and usable method of clinical outcome assessment compared with established measures of behavior and mood. Concerns have been raised around attrition in and adherence to these new protocols, particularly over the medium to long term by people with mood disorders.

**Objective:**

This systematic review and meta-analysis assessed attrition from and adherence to active and passive ambulatory assessment and mood monitoring protocols by people with bipolar disorder and depression over the medium and long term.

**Methods:**

Randomized controlled trials and nonrandomized studies were identified and rated for risk of bias. Adherence and attrition data were pooled to calculate effect sizes. We analyzed specific factors that we hypothesized a priori could affect the prevalences of attrition and adherence by means of subgroup meta-analysis or metaregression modeling.

**Results:**

We evaluated 77 mood tracking or ambulatory assessment studies including 17,123 participants. Pooled adherence was 0.64% (95% CI 0.59%-0.70%; *P*<.001), and pooled attrition was 0.28% (95% CI 0.22%-0.34%; *P*<.001). Three factors had a statistically significant subgroup difference for adherence: The presence of financial incentives increased adherence, and the presence of mood monitoring reminders and a higher study risk of bias decreased adherence. Four factors had a statistically significant subgroup difference for attrition: Digital mood monitoring decreased attrition versus analogue studies, but mood monitoring reminders, mood monitoring versus other protocols, and a high risk of study bias increased attrition. These analyses, however, were vulnerable to confounding by study design and protocol design. Attrition rates were not reported by 17 studies (17/77, 22%), and 20 studies (20/77, 26%) did not report adherence rates. Most studies had a low-to-moderate risk of bias, but heterogeneity was very high. Only 16 studies reported adherence systematically.

**Conclusions:**

Reporting of attrition and adherence to ambulatory assessments was not systematic nor universal, and until it is, analyses are unlikely to demonstrate clear conclusions. We found very high heterogeneity and evidence of publication bias, and this limited the certainty of our conclusions. Financial incentives may increase adherence, and attrition may be lower in digital than analogue studies of mood monitoring. There was no statistically significant difference in adherence and attrition between studies of passive and active ambulatory assessments. Reminders of mood monitoring increased attrition and decreased adherence, but the results may be confounded by longer length of follow-up versus other studies.

## Introduction

Technological developments, such as wearables and smartphone sensors, have enhanced the capacity for mood monitoring through both active (manual input) and passive (automated) data collection [1]. Although these tools offer promising applications in mood disorders (particularly for addressing the limitations of traditional measurement tools for capturing rapidly shifting mood change [2]), smartphone-based ambulatory assessments may pose specific risks and challenges, especially for vulnerable populations [3]. The definition of ambulatory assessment encompasses a wide range of methods that leverage mobile technologies to repeatedly study individuals—often in real time and in their natural environments [4]. This includes mood monitoring (repeatedly tracking one’s mood over time), remote measurement technologies (defined as wearable devices that passively collect data without any active user input), and ecological momentary assessment (EMA), which is defined as a type of assessment involving more intensive, frequent self-reports throughout the day in real time and “in the moment” [5].

This review explored dropout, attrition, and adherence associated with mood monitoring, mood tracking, and ambulatory assessment by individuals with unipolar depression and bipolar disorder. This study focused on studies exploring mood monitoring (defined as regularly tracking and appraising one’s mood), and many of the studies included here also fall under the similar categories of EMA, remote measurement technologies, or ambulatory assessment depending on the frequency, manner, and technology used to track mood. There is also considerable overlap between these definitions (defined in the previous paragraph). Here, mood monitoring was used both as an intervention (in randomized and nonrandomized studies) and as a method for measuring outcomes (again in randomized and nonrandomized studies).

Because of this, this study focused specifically on individuals with unipolar depression and bipolar disorder. This is due to the evidence suggesting that this group faces distinct risks and challenges that may cause issues with attrition and adherence [6,7]. Mood disorders represent promising conditions for mood monitoring, as they are characterized by subtle mood shifts over time that may respond well to early intervention. They also represent a large global source of disability with depression as the leading cause [8]. Both people with depression and people with bipolar disorder (particularly those who experience psychosis) may have particularly poor adherence rates; therefore, improving this may be vital to realizing some of the benefits that ambulatory assessment approaches could offer for understanding and treating these conditions [9,10].

Due to the perceived burden of some ambulatory assessment protocols [6], concerns have been raised about their usability and acceptability, particularly over longer periods of time in clinical populations [9]. Therefore, this study focused on 3 months or longer. There are likely to be factors that could be adjusted to optimize usability and acceptability and thus adherence and attrition. This could improve the overall quality of the data collected and thus improve the research or interventions that derive from the data. Optimizing adherence and attrition is also of benefit to clinical research and could considerably decrease sample sizes required for future ambulatory assessment research [11].

Ambulatory assessment protocols are highly heterogenous and, even within the same discipline, use a wide variety of different methodologies [5]. These vary in terms of the type and frequency of assessment schedule as well as the technology used to collect the data and whether the assessment is also used as an intervention. Because of this heterogeneity, previous reviews have attempted to untangle which factors affect adherence and attrition, but these have only been in short-term use rather than medium or long-term assessment. This medium or long-term use may be necessary to evaluate the fluctuations in mood that occur over time, particularly in depression and bipolar disorder [9,11]. Longer-term data are likely to be fundamental for digital phenotyping and offering personalized psychiatry, which could, for example, identify early warning signs and prevent or predict relapse [12], aims that are particularly relevant for mood disorder research [13,14].

There are several reasons why adherence to long-term ambulatory assessment use may be different to that of shorter protocols. Ambulatory assessment protocol adherence tends to decrease over the duration of the assessment and by people with higher levels of negative affect (eg, mood disorders), but again, this has not been assessed over longer time frames [9,15]. Previous work has identified that studies can improve adherence by offering financial incentives, minimizing the quantity of assessments, and the assessments occurring at a prespecified time [9,11]. Other studies have not identified any significant predictors of attrition [16]. It remains unclear if these factors also apply to medium or long-term approaches.

This review aimed to assess attrition and adherence over the medium and long term to ambulatory assessment and mood monitoring protocols and consider the factors that may affect these (eg, diagnosis, financial incentives, remote or in-person enrollment, reminders to complete the mood assessment, frequency of mood assessment).

## Methods

We used a Cochrane Handbook for Systematic Reviews of Interventions–based methodology. We completed a PRISMA (Preferred Reporting Items for Systematic Reviews and Meta-Analysis) checklist. The study was pre-registered in accordance with the International Prospective Register of Systematic Reviews (PROSPERO: CRD42023396473 [17]).

### Inclusion Criteria

Included studies met the following criteria: included self-monitoring, mood monitoring, or repeated symptom assessment with people with bipolar disorder or depression using an interventional or observational design over a minimum period of 3 months, with rating of symptoms weekly at a minimum. Operationally, for this review, we defined this repeated symptom assessment as either mood monitoring or ambulatory assessment (as defined in the previous section—with all mood monitoring conducted as an ambulatory assessment but not all ambulatory assessment being mood monitoring). We chose this broad definition because many ambulatory assessment protocols have the potential to be used for mood monitoring as an intervention or for research outcomes, even if this was not the intended aim of the study, although all included studies used the repeated symptom assessment either as an intervention or research outcome.

The studies should have either used a validated measure of mood or validated the chosen measure with a validated mood measure. The studies could be published in any language and could be digital or nondigital, although we acknowledged that the majority of studies would utilize digital technologies. We included nonrandomized or randomized studies with 20 or more participants with bipolar disorder or depression [18]. We searched the gray literature (eg, conference abstracts, dissertations, policy literature, reports via ProQuest and Google Scholar—full details are in the next section) for unpublished studies that were eligible for inclusion.

### Search Strategy and Selection Criteria

The complete search strategy is listed in Multimedia Appendix 1. We searched Ovid MEDLINE, EMBASE, PsychINFO, SCOPUS, IEE Xplore, Proquest SciTech Collection, Proquest dissertations and theses global, and Google Scholar using the search terms. The initial search was conducted on March 3, 2023, and updated on October 28, 2024. All abstracts were appraised by 2 independent screeners, any disagreements were discussed, and a consensus was reached, with adjudication by a third independent screener if required. We acquired the full text of any potentially relevant papers, and if we were unable to source the full text of the study, we then contacted the corresponding author to request the paper. To determine if potentially relevant studies met the inclusion criteria, the full text was reviewed separately by 2 authors, again with discussion and consensus with a third reviewer if necessary. All papers for inclusion were reference checked along with relevant systematic reviews [19-28]. Key authors were also emailed to see if any ongoing unpublished studies could be included.

### Data Extraction

Data were extracted by 2 independent reviewers from studies meeting the inclusion criteria using identical data extraction forms. Irregularities in the data extraction were discussed, and any discrepancy was resolved with discussion.

In this review, we defined attrition as the number of participants who had withdrawn from the study and no longer contributed data after randomization or baseline assessment. We defined adherence as the number of ambulatory assessments completed by those who remained in the study. When calculating attrition and adherence, we included anyone randomized into the trial, even if they did not subsequently complete any or many mood assessments. This was because preliminary analyses showed the reporting of the exact number of people who took part in the mood monitoring was poor, and our aim was to create pragmatic attrition and adherence values that would be useful to the power calculations of future ambulatory assessment studies. Using the number randomized was likely to affect the calculated estimates and was likely to increase the attrition rate by increasing the denominator size. For example, this may have included individuals without any actual use of the monitoring tool but who were randomized to the intervention arm. Even if not reflective of the adherence and attrition of highly motivated individuals, nonetheless, this is to give a pragmatic figure for the design of future studies. This estimate then gave a pragmatic figure for the design of future studies that use an optimal intention-to-treat design rather than estimates based on selection of more motivated and adherent participants.

Attrition and adherence were calculated using what we judged to be the most systematic and pragmatic metric available (eg, the longest follow-up available using systematically acquired data). Not all studies reported raw figures, and some only reported attrition %, which we included when available. For only a minority of studies, adherence was reported systematically using device-generated data. Many studies did not report this, and some studies reported the number of participants completing the study as the adherence: We included these less accurate metrics if there was not a more systematic metric reported. For many of these metrics, we were required to email the authors to request the attrition and adherence data due to no reporting in the manuscript. For attrition, using the number randomized as the denominator may have underestimated the attrition, as many of these individuals would have never used the ambulatory assessment protocol. On the other hand, including some nonsystematically gathered data likely distorts the pooled estimate (possibly by underestimating attrition and adherence but this is not certain), but we did, however, control for this in a sensitivity analysis documented in the following sections.

### Assessment of Study Bias

For observational studies, the Cochrane Collaboration tool for assessing the risk of bias in nonrandomized studies of interventions [29] was used. For randomized controlled trials (RCTs), we used the Cochrane risk of bias tool for randomized trials for each study [30]. Risk of bias was assessed by 2 independent reviewers, and any disagreement was resolved via discussion.

### Synthesis of Results

We grouped studies together, where possible, according to the variable assessed (eg, adherence or attrition) and pooled the data in a meta-analysis. We conducted subgroup analysis on specific factors that we hypothesized a priori could affect the prevalences of attrition and adherence. Results of each primary study were pooled using the inverse variance weighted approach with a random or fixed effects model, informed by examining the between-studies heterogeneities. Stata metan code was used to perform the analysis for proportion data, and metareg code was used for metaregression modeling.

## Results

### Search Results

**Figure 1.**
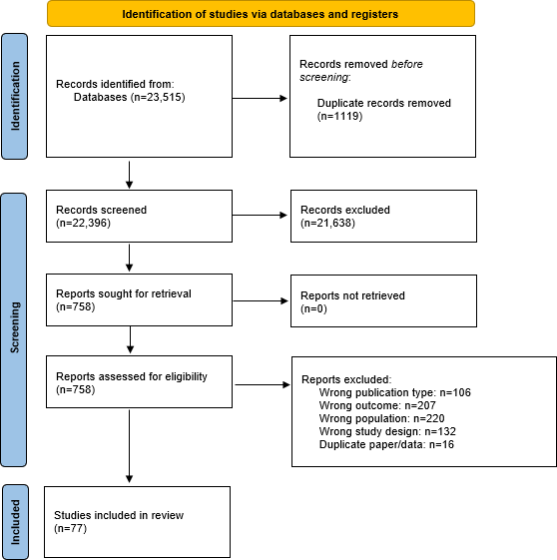
PRISMA (Preferred Reporting Items for Systematic Reviews and Meta-Analysis) flow diagram of included studies.

The search identified 23,515 papers (Figure 1). No studies not published in English met the inclusion criteria. Following title and abstract screening, 21,638 articles were excluded, resulting in a total of 758 papers being reviewed in full. A total of 77 papers met the eligibility criteria and were included in the review. The 77 studies included 17,123 participants: 17 studies did not report attrition rates, 20 studies did not report adherence rates, 34 studies did not report if financial incentives were offered, and 25 studies did not report the recruitment method. The average mood tracking or ambulatory assessment duration was 6.9 months. Reporting of adherence was not universal nor systematic: Of the 57 studies that reported adherence, 34 studies did not specify how the adherence statistic was calculated, and 23 systematically used device-generated data to calculate this.

Tables S1 and S2 in Multimedia Appendix 2 display the detailed characteristics of the studies and the mood monitoring protocols used. These tables are stratified by clinical condition and type of study (eg, randomized or nonrandomized).

We included mixed samples if they included 20 or more participants with either depression or bipolar disorder. In the depression studies, this included 899 participants with primary diagnoses other than depression or healthy controls of a total of 9310 participants included. In bipolar disorder studies, there were 83 participants with primary diagnoses other than bipolar disorder or healthy controls of a total of 7813 included. In total, 5.7% (977/17,123) of the total participants did not have a primary diagnosis of depression or bipolar disorder.

We included relapse prevention or maintenance trials, as this is a core element of treatment of bipolar disorder and depression, which are usually relapsing and remitting illnesses. With studies of variable follow-up durations, we included the attrition or adherence from the latest available outcome, and this was a pragmatic decision around the design of future studies that will require long-term follow-ups due to the lifelong nature of many mood disorders.

### Risk of Bias Assessments

The methodological quality of these studies was variable, but most were considered of low-to-moderate risk of bias (Table S3 in Multimedia Appendix 2).

### Meta-Analysis

As planned a priori, we conducted a subgroup analysis of 5 different factors for both adherence and attrition reported in Table S1 in Multimedia Appendix 2: type of mood monitoring, mood disorder, RCT versus nonrandomized study, digitization of mood monitoring, presence of reminders to complete the mood monitoring, in-person or remote enrollment, and the presence of financial incentives.

**Figure 2.**
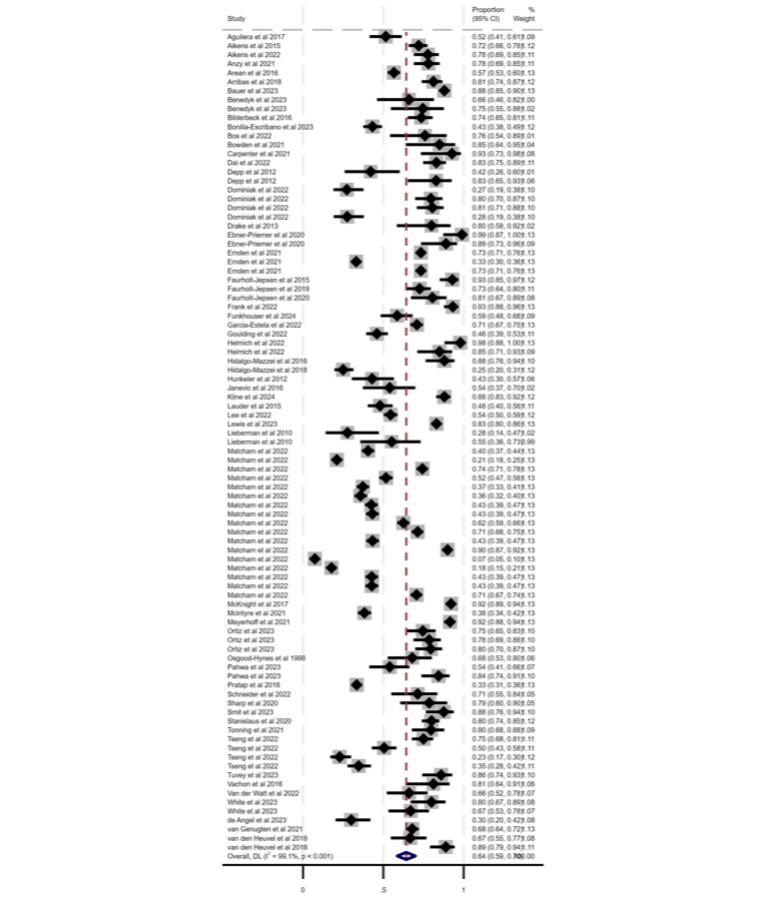
Forest plot of the prevalence of adherence with percentage weighting [31-96].

Meta-analytic pooling of adherence (0.64%, 95% CI 0.59%-0.70%; *P*<.001) and attrition (0.28%, 95% CI 0.22%-0.34%; *P*<.001) is reported in Figures2  3, respectively. For adherence, heterogeneity was high (Cochran *Q*_90_=10,052.49; *P*<.001; *I*^2^=99.1%). Likewise for attrition, heterogeneity was high (Cochran *Q*_73_=6615.17; *P*<.001; *I*^2^=98.9%). Because of the pronounced heterogeneity, we used a random effects model to pool the data. Both of the funnel plots for the total prevalences of adherence and attrition demonstrated visual asymmetry and thus suggested possible publication bias or small study effects in the meta-analytic estimate (Figures S1 and S3 in Multimedia Appendix 2). The Egger test demonstrated that there were no small-study effects (Figures S2 and S4 in Multimedia Appendix 2) for both the adherence and attrition analyses.

**Figure 3.**
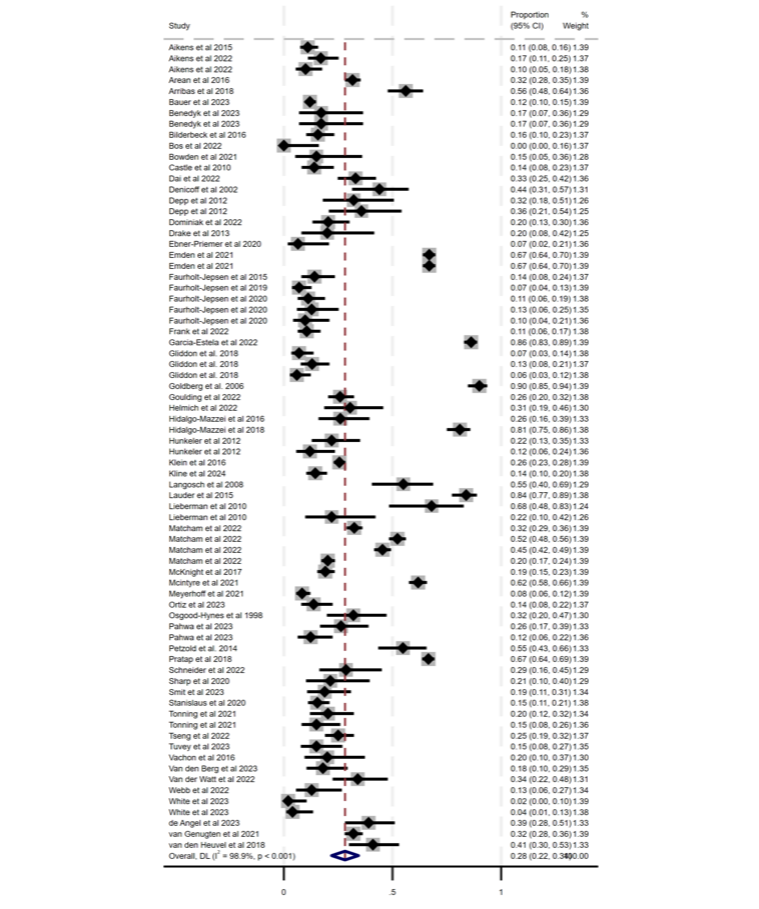
Forest plot of the prevalence of attrition with percentage weighting [31-41,43,44,46-52,54-57,59-68,70-76,78-82,85-96].

We report subgroup analyses in Figures S5-S16 in Multimedia Appendix 2. The interpretation of any differences here should not be interpreted causally due to the limitations of the data; likewise, any notable absence of effect should not be interpreted as conclusive equivalence due to potential power limitations. Only 3 factors had a statistically significant subgroup difference for adherence: the presence of financial incentives, mood monitoring reminders, and study risk of bias. Studies without financial incentives had lower adherence (0.65%, 95% CI 0.52%-0.79%) than those offering financial incentives (0.77%, 95% CI 0.66%-0.88%; between-group *Q*_2_=6.38; *P*=.04). There was not a statistically significant effect of financial incentives on attrition, however: studies without financial incentives: 0.22% (95% CI 0.07%-0.37%); studies with financial incentives: 0.21% (95% CI 0.10%-0.31%; between-group *Q*_2_=3.91; *P*=.14). Studies with mood monitoring reminders had lower adherence (0.61%, 95% CI 0.54%-0.67%) than studies without mood monitoring reminders (0.78%, 95% CI 0.69%-0.88%; between-group *Q*_2_=11.25; *P*=.004).

A statistically significant subgroup difference was also not present for adherence for studies that systematically reported adherence via device-level data compared with those that did not: studies that did not systematically report adherence: 0.67% (95% CI 0.59%-0.75%); studies that systematically reported adherence: 0.62% (95% CI 0.54%-0.69%; between-group *Q*_1_=1*; P*=.32).

A statistically significant subgroup difference for attrition was present for 5 factors: presence of mood monitoring reminders, digital vs analogue mood monitoring, mood monitoring study or non-mood monitoring control, and study risk of bias. Studies with mood monitoring reminders had higher attrition (0.30%, 95% CI 0.22%-0.37%) than studies without mood monitoring reminders (0.27%, 95% CI 0.16%-0.38%; between-group *Q*_2_=56.80; *P*<.001). Analogue studies had higher attrition (0.40%, 95% CI 0.16%-0.64%) than digital studies (0.28%, 95% CI 0.21%-0.35%; between-group *Q*_2_=29.66; *P*<.001). Mood monitoring studies had higher attrition (0.29%, 95% CI 0.23%-0.36%) than non-mood monitoring control groups (0.09%, 95% CI 0.06%-0.12%; between-group *Q*_2_=31.94; *P*<.001).

There is likely an element of confounding by study design and protocol burden in these subgroup analyses, as studies with EMA reminders (n=23) reported a longer mean follow-up duration (mean 11.02, SD 8.5 months) than studies without EMA reminders (n=86; mean 7.48, SD 4.22 months).

There was no statistically significant difference in adherence nor attrition between active and passive mood monitoring (Figures S5 and S12 in Multimedia Appendix 2), in-person versus remote enrollment or recruitment (Figures S11 and S18 in Multimedia Appendix 2), and depression versus bipolar disorder (Figures S6 and S13 in Multimedia Appendix 2).

There were statistically significant subgroup differences in both adherence and attrition between studies deemed to have a high risk of bias (≤4 areas of low risk of bias as classified on the Cochrane risk of bias tool) versus those deemed to have a low risk of bias (5 or 6 areas of low risk of bias). High-risk studies had lower adherence (0.60%, 95% CI 0.53%-0.68%) than low-risk studies (0.70%, 95% CI 0.64%-0.76%; between-group *Q*_1_=3.93; *P*=.047). High-risk studies had higher attrition (0.38%, 95% CI 0.29%-0.46%) than low-risk studies (0.19%, 95% CI 0.14%-0.24%; between-group *Q*_1_=13.75; *P*<.001).

### Metaregression

As the heterogeneity was very high, we conducted a metaregression to assess potential sources of this heterogeneity. We explored the effects of financial incentives, ambulatory assessment reminders, digital or analogue protocol, active or passive ambulatory assessment, gender, age, and ambulatory assessment duration on heterogeneity. The residual heterogeneity remained very high despite this, and the full results are reported in Table S4 in Multimedia Appendix 2.

Notably, active ambulatory assessments had a statistically significant higher attrition than nonambulatory assessment–based controls (odds ratio [OR] 1.23, 95% CI 1.00-1.51; *P*=.046). However, attrition rates for studies using both active and passive ambulatory assessments were not statistically significant compared with the control group (OR 1.14, 95% CI 0.92-1.42; *P*=.23). Likewise, attrition rates for passive ambulatory assessment studies were not statistically significantly different than the control groups (OR 1.23, 95% CI 0.97-1.57; *P*=.09). This suggests studies using just active ambulatory assessment measures may have a uniquely high burden for participants.

There was a statistically significant negative effect of ambulatory assessment duration on adherence rates (OR 0.99, 95% CI 0.99-1.00; *P*=.01), and adherence declined as assessment duration increased.

There was a statistically significant effect of the number of low-risk domains in a study leading to lower attrition (OR 0.94, 95% CI 0.90-0.98; *P*=.002) but not adherence (OR 1.02, 95% CI 0.98-1.07; *P*=.26).

## Discussion

### Summary of Findings

This meta-analysis examined long-term adherence and attrition in mood monitoring or ambulatory assessment studies by people with depression and bipolar disorder and, to our best knowledge, is the first review to do so. We documented larger improvements in attrition in digital studies than in analogue studies, in adherence in studies with financial incentives versus nonfinancial incentives, and increased attrition or decreased adherence in studies with mood monitoring reminders versus those without reminders. There was also improved adherence or attrition in higher-quality studies. Crucially, there were no differences in adherence and attrition between studies of passive and active mood monitoring, although active ambulatory assessments had higher attrition than non-mood monitoring controls. However, we noted there was a very high level of heterogeneity in the meta-analysis, with some evidence of publication bias, which may mean that studies showing no difference in attrition or adherence were not necessarily reported, and no clear picture has emerged from our results. The high heterogeneity was not due to the reasons explored in the metaregression: age, gender, assessment duration, active or passive assessment, digital or analogue, or assessment reminders or financial incentives. Reporting of adherence and attrition was not universal nor systematic, and until it is, analyses are unlikely to provide any clear conclusions.

### Context of Findings

The mood monitoring adherence statistics we report here (many of which were EMA studies) are lower than those in studies of dropout and adherence with EMA protocols in both clinical and nonclinical populations [11,97-99]. They are also lower than the recommended rate of 80% for EMA studies [100]. Missing data, particularly higher than suggested recommended rates, can limit the conclusions that are drawn from statistical inference and have a significant effect on the study’s statistical power [98]. Furthermore, research suggests that missing data in EMA studies are not at random and thus more likely to introduce nonresponse bias—for example, participants are more likely to miss certain assessments at specific times (eg, when delivering childcare at a specific time in the evening) [101]. The rates of adherence reported here are thus concerning to the methodological validity of these mood monitoring studies, some of which are also EMA studies. This is further demonstrated by the higher attrition in mood monitoring groups when compared with non-mood monitoring controls, raising the question of whether mood monitoring is itself aversive.

The follow-up length that we assessed was also much longer than other studies and other reviews, and this may partially explain the lower adherence. Here, the average mood monitoring or ambulatory assessment protocol length was approximately 7 months, while many reviews have only assessed adherence over 7 days. Long-term studies generally demonstrate higher attrition than short-term studies [102]. Furthermore, clinical populations may have higher attrition rates and lower adherence than nonclinical populations. This is particularly true of people with mood disorders who can struggle with drive, motivation, and organization. The attrition rate was comparable to other studies of bipolar disorder [103] or depression and clinical trials of smartphone apps [104]. This was reassuring considering the increased participant burden reported in qualitative studies and suggests that mood monitoring studies, with regard to attrition and adherence, may not be particularly different or uniquely stressful than studies using established measures of assessment.

### Protocol Design

Crucially, we did not demonstrate better adherence and attrition in passive mood monitoring studies than with active protocols. This was in contrast to qualitative research suggesting passive mood monitoring is preferred, as it is perceived as being less intrusive (and has less participation burden) by people with bipolar disorder and depression [6]. This may be due to people not adhering to or dropping out from mood monitoring studies for reasons other than the frequent completion of self-report measures. Some of these concerns driving nonadherence and dropout may be around data security, not feeling that they themselves are receiving a benefit, or the presence of adverse effects, as opposed to the type of mood monitoring or the way this is delivered [6,105].

Financial incentives did not appear to affect attrition but were associated with improved adherence. This was in keeping with previous research with similar findings [11], finding 12% higher adherence if financial incentives were given (77%) compared with when financial incentives were not given (65%). This is a very large improvement in adherence, particularly over many months. There are, however, concerns that paying individuals to participate in research may exert undue influence and coercion [106] and potentially select individuals who are more motivated by the financial reward than the potential for digital technology to improve their mental disorder via mood monitoring or to contribute to research via ambulatory assessment—both of which likely require motivation and commitment [6]. This may thus create selection biases. This also may be especially problematic in studies where reward responsivity is being measured since financial incentives might be perceived as a reward and therefore a confounder. Participants should also be given the choice to not accept a financial reward if they choose; some people find such rewards coercive or forming an obligation when they wish to take part from free will.

Mood monitoring reminders were associated with increased attrition and decreased adherence. It is possible that a higher number of notifications and reminders increases participant burden and leads to dropout by some individuals or that those studies with more burdensome protocols also include reminders. There is likely an element of confounding by study design and protocol burden in these analyses. Furthermore, this is in contrast to other findings, where increased participant burden (eg, with active mood monitoring) was not associated with an increased dropout versus passive mood monitoring (which is often designed to minimize this burden). One possible explanation for the lack of difference in dropout despite an increased participant burden is that active mood monitoring directly asks the user their opinion and makes them aware of their mood versus passive monitoring, which sits in the background and provides no immediate feedback to the participant. This active feedback-driven process may compensate for the increased burden.

### Reporting Standards

Reporting of attrition (60/77, 78% of studies) and adherence (57/77, 74% of studies) was low, suggesting that studies should report these metrics routinely, particularly considering the importance of these metrics to mood monitoring data validity. The number of studies reporting these metrics in the published manuscripts was even lower; we contacted a large number of authors individually to find much of this information, which was often difficult to locate in the first instance. Only a small minority of studies reported adherence in a systematic way from device data—most did not clarify the nature of the data they reported (eg, self-report adherence, objective app data).

Researchers have previously produced important guidelines on the reporting of mood monitoring data (eg, eMOOD guidelines [26]). Although these do report the importance of good adherence data, they do not mention the systematic underreporting of adherence and attrition statistics and provide no factual basis for the statement that adherence to mood monitoring is often low. The quantified evidence summarized here provides a basis for specific recommendations around the reporting of adherence and attrition data, which has previously been sparse. We suggest that preference be given to systematically collected device-level data that allow direct comparison between protocols. Acceptable attrition and adherence thresholds should be prespecified based on power calculations and the intended purpose of the ambulatory assessment. Power calculations for sample size should consider what effects acceptable attrition and adherence might have on outcome; power calculations without these considerations might then be considered incomplete and probably underpowered.

The reporting of attrition should be in accordance with CONSORT (Consolidated Standards of Reporting Trials) for randomized studies [107] and alternatives for nonrandomized studies [108]. This will allow researchers to assess attrition based on the number of participants enrolled or those with actual use of the ambulatory assessment, as these figures are likely to tell us different things and inform future studies in different ways. Researchers should report attrition based on the number of participants enrolled as an intention-to-treat analysis, as this will generate a more generalizable result for clinical practice. They should also report a per-protocol analysis of those who attempted to use the assessment as intended, as this will give an indication of attrition by those motivated and willing to undertake these assessments at the beginning of the study. These align with current guidelines for interventions in general in RCTs and nonrandomized studies. We make further broader recommendations incorporating a broad range of qualitative and quantitative evidence that puts poor adherence and attrition reporting in the wider ambulatory assessment or mood monitoring context [109].

Lower risk-of-bias studies had considerably less attrition and higher adherence. Higher-quality studies may be more rigorously conducted, and this may lead to improved follow-up rates and more participant-friendly protocols (eg, via more qualitative development of the protocol or lived experience input) to which it is easier to adhere. Therefore, the overall risk of bias of a study may be a marker of a higher-quality study that is less onerous for participants. These attrition and adherence results, even for high-quality studies, suggest that there is a high level of attrition and low level of adherence to data collection that are hard to address with methods that rely on longer-term active data collection alone. Further investigation on the optimal mix of methods of digital collection (eg, active and passive with optimal methods of support and reward) is merited .

### Participant Engagement

Future mood monitoring studies with mood disorders should prioritize consistent reporting of adherence and attrition and use financial incentives to support participant engagement. This study failed to find a multitude of specific factors that improve attrition and adherence, aside from financial incentives and reminders (which surprisingly had a negative effect on adherence and attrition). This thus raises questions about additional methods of improving the acceptability and usability of these protocols. Many of these factors are likely to be protocol specific and will likely require lived experience input, qualitative research, and ethical implementation to create a protocol that has high acceptability and usability, particularly with long-term studies. Some mood monitoring protocols reported attrition as high as 92%, and others reported adherence as low as 18%. It remains unclear what is a realistic attrition or adherence rate for mood monitoring studies with people with mood disorders, and this may rarely reach the recommended 80%, particularly for individuals with a higher severity of illness.

### Strengths and Limitations

The strengths of this review are that we evaluated studies using mood monitoring over a minimum period of 3 months. We argue that 3 months of follow-up was required to accurately assess mood, attrition, and adherence in a clinically meaningful way to assist the design of medium or long-term trials in the future. The average duration of follow-up was much longer than other reviews—approximately 7 months—a more clinically material length of time. We did not include many mood monitoring procedures that utilized shorter follow-up and are further away from implementation as interventions or as research tools. This allows us to make clear conclusions about mood monitoring methods that have the ability to affect the current delivery of mental health trials. One limitation of this review is that the vast majority of trials had issues with risk of bias, specifically around blinding of the intervention or mood monitoring procedure, but this is an issue with trials of psychological treatments more broadly where perfect blinding is not possible. We were also not able to include all studies in the meta-analysis due to underreporting of key data, which is in itself both a limitation and an important finding highlighting the lack of standardization in the reporting of key mood monitoring metrics. Furthermore, there was large statistical heterogeneity when aggregating data, and this reflects the heterogeneity of the wide-ranging mood monitoring protocols (eg, type of mood disorder, population, duration of mood monitoring, and intensity of mood monitoring); despite using a random-effects model, this may still limit the generalizability of our findings, mask important contextual differences, and limit the comparability of the summary estimates across studies. In addition, our analyses should not be interpreted causally due to these limitations, and they were vulnerable to confounding by study design and protocol burden.

### Conclusions

To conclude, this meta-analysis examined long-term adherence and attrition in mood monitoring studies by people with mood disorders. Attrition and adherence were lower for people with bipolar disorder and depression than for other nonclinical or clinical populations but comparable to other non-mood monitoring studies with people with mood disorders. We demonstrated that some key factors may improve adherence and attrition (crucially, there were no differences in adherence and attrition between studies of passive and active mood monitoring), but the certainty of our findings is limited due to the lack of systematic and universal reporting of adherence and attrition in ambulatory assessment studies. These findings should inform the design of future mood monitoring protocols—prioritizing systematic and universal reporting of adherence and attrition—and be interpreted alongside qualitative findings to optimize real-world acceptability and utility. Until reporting of adherence and attrition improves, further analyses are unlikely to provide any clear conclusions.

## Supplementary material

10.2196/83765 Multimedia Appendix 1 Search strategy

10.2196/83765 Multimedia Appendix 2 Study characteristics and risk of bias assessments.

10.2196/83765 Checklist 1 PRISMA (Preferred Reporting Items for Systematic Reviews and Meta-Analysis) checklist.

## Abbreviations

CONSORT: Consolidated Standards of Reporting Trials
EMA: ecological momentary assessment
OR: odds ratio
PRISMA: Preferred Reporting Items for Systematic Reviews and Meta-Analysis
RCT: randomized controlled trial

## References

[1] Matcham F, Barattieri di San Pietro C, Bulgari V (2019). Remote assessment of disease and relapse in major depressive disorder (RADAR-MDD): a multi-centre prospective cohort study protocol. BMC Psychiatry.

[2] Gromatsky M, Sullivan SR, Spears AP (2020). Ecological momentary assessment (EMA) of mental health outcomes in veterans and servicemembers: a scoping review. Psychiatry Res.

[3] Linardon J, Fuller-Tyszkiewicz M (2020). Attrition and adherence in smartphone-delivered interventions for mental health problems: a systematic and meta-analytic review. J Consult Clin Psychol.

[4] Trull TJ, Ebner-Priemer U (2013). Ambulatory assessment. Annu Rev Clin Psychol.

[5] aan het Rot M, Hogenelst K, Schoevers RA (2012). Mood disorders in everyday life: a systematic review of experience sampling and ecological momentary assessment studies. Clin Psychol Rev.

[6] Astill Wright L, Majid M, Moore M (2025). The user experience of ambulatory assessment and mood monitoring in bipolar disorder: systematic review and meta-synthesis of qualitative studies. J Med Internet Res.

[7] van Genugten CR, Schuurmans J, Lamers F (2020). Experienced burden of and adherence to smartphone-based ecological momentary assessment in persons with affective disorders. J Clin Med.

[8] Friedrich MJ (2017). Depression is the leading cause of disability around the world. JAMA.

[9] Vachon H, Viechtbauer W, Rintala A, Myin-Germeys I (2019). Compliance and retention with the experience sampling method over the continuum of severe mental disorders: meta-analysis and recommendations. J Med Internet Res.

[10] Sokolovsky AW, Mermelstein RJ, Hedeker D (2014). Factors predicting compliance to ecological momentary assessment among adolescent smokers. Nicotine Tob Res.

[11] Wrzus C, Neubauer AB (2023). Ecological momentary assessment: a meta-analysis on designs, samples, and compliance across research fields. Assessment.

[12] Baumeister H, Montag C (2019). Digital Phenotyping and Mobile Sensing: New Developments in Psychoinformatics.

[13] Morriss RK, Faizal MA, Jones AP, Williamson PR, Bolton C, McCarthy JP (2007). Interventions for helping people recognise early signs of recurrence in bipolar disorder. Cochrane Database Syst Rev.

[14] Morriss RK, Bolton CA, McCarthy JP, Marshall M, Williamson PR, Jones AP (2018). Interventions for helping people recognise early signs of recurrence in depression. Cochrane Database Syst Rev.

[15] Fuller-Tyszkiewicz M, Skouteris H, Richardson B, Blore J, Holmes M, Mills J (2013). Does the burden of the experience sampling method undermine data quality in state body image research?. Body Image.

[16] Bell IH, Eisner E, Allan S (2024). Methodological characteristics and feasibility of ecological momentary assessment studies in psychosis: a systematic review and meta-analysis. Schizophr Bull.

[17] Astill Wright L, Morriss R (2023). Mood-tracking and self-monitoring in bipolar disorder and depression. PROSPERO.

[18] (2021). Bipolar Disorder (Update) – Review Protocols. National Institute for Health and Care Excellence.

[19] Antosik-Wójcińska AZ, Dominiak M, Chojnacka M (2020). Smartphone as a monitoring tool for bipolar disorder: a systematic review including data analysis, machine learning algorithms and predictive modelling. Int J Med Inform.

[20] Dubad M, Winsper C, Meyer C, Livanou M, Marwaha S (2018). A systematic review of the psychometric properties, usability and clinical impacts of mobile mood-monitoring applications in young people. Psychol Med.

[21] Palmier-Claus J, Lobban F, Mansell W (2021). Mood monitoring in bipolar disorder: Is it always helpful?. Bipolar Disord.

[22] Faurholt-Jepsen M, Munkholm K, Frost M, Bardram JE, Kessing LV (2016). Electronic self-monitoring of mood using IT platforms in adult patients with bipolar disorder: a systematic review of the validity and evidence. BMC Psychiatry.

[23] van der Watt ASJ, Odendaal W, Louw K, Seedat S (2020). Distant mood monitoring for depressive and bipolar disorders: a systematic review. BMC Psychiatry.

[24] Ortiz A, Maslej MM, Husain MI, Daskalakis ZJ, Mulsant BH (2021). Apps and gaps in bipolar disorder: a systematic review on electronic monitoring for episode prediction. J Affect Disord.

[25] Malhi GS, Hamilton A, Morris G, Mannie Z, Das P, Outhred T (2017). The promise of digital mood tracking technologies: are we heading on the right track?. Evid Based Mental Health.

[26] Faurholt-Jepsen M, Geddes JR, Goodwin GM (2019). Reporting guidelines on remotely collected electronic mood data in mood disorder (eMOOD)-recommendations. Transl Psychiatry.

[27] Wenze SJ, Miller IW (2010). Use of ecological momentary assessment in mood disorders research. Clin Psychol Rev.

[28] Koenders MA, Nolen WA, Giltay EJ, Hoencamp E, Spijker AT (2015). The use of the prospective NIMH Life Chart Method as a bipolar mood assessment method in research: a systematic review of different methods, outcome measures and interpretations. J Affect Disord.

[29] Sterne JA, Hernán MA, Reeves BC (2016). ROBINS-I: a tool for assessing risk of bias in non-randomised studies of interventions. BMJ.

[30] Higgins JPT, Altman DG, Gøtzsche PC (2011). The Cochrane Collaboration’s tool for assessing risk of bias in randomised trials. BMJ.

[31] Hidalgo-Mazzei D, Mateu A, Reinares M (2016). Psychoeducation in bipolar disorder with a SIMPLe smartphone application: feasibility, acceptability and satisfaction. J Affect Disord.

[32] van den Heuvel S, Meije D, Regeer EJ, Sinnema H, Riemersma RF, Kupka RW (2018). The user experiences and clinical outcomes of an online personal health record to support self-management of bipolar disorder: a pretest-posttest pilot study. J Affect Disord.

[33] Van der Watt ASJ, Dalvie N, Seedat S (2022). Weekly telephone mood monitoring is associated with decreased suicidality and improved sleep quality in a clinical sample. Psychiatry Res.

[34] Drake G, Csipke E, Wykes T (2013). Assessing your mood online: acceptability and use of Moodscope. Psychol Med.

[35] Hidalgo-Mazzei D, Reinares M, Mateu A (2018). OpenSIMPLe: a real-world implementation feasibility study of a smartphone-based psychoeducation programme for bipolar disorder. J Affect Disord.

[36] García-Estela A, Cantillo J, Angarita-Osorio N (2022). Real-world implementation of a smartphone-based psychoeducation program for bipolar disorder: observational ecological study. J Med Internet Res.

[37] de Angel V, Adeleye F, Zhang Y (2023). The feasibility of implementing remote measurement technologies in psychological treatment for depression: mixed methods study on engagement. JMIR Ment Health.

[38] Osgood-Hynes DJ, Greist JH, Marks IM (1998). Self-administered psychotherapy for depression using a telephone-accessed computer system plus booklets. J Clin Psychiatry.

[39] Sharp KJ, South CC, Chin Fatt C, Trivedi MH, Rethorst CD Pilot studies to evaluate feasibility of a physical activity intervention for persons with depression. J Sport Exerc Psychol.

[40] Pahwa M, McElroy SL, Priesmeyer R (2024). KIOS: a smartphone app for self-monitoring for patients with bipolar disorder. Bipolar Disord.

[41] Bowden CL, Priesmeyer R, Tohen M (2021). Development of a patient-centered software system to facilitate effective management of bipolar disorder. Psychopharmacol Bull.

[42] Janevic MR, Aruquipa Yujra AC, Marinec N (2016). Feasibility of an interactive voice response system for monitoring depressive symptoms in a lower-middle income Latin American country. Int J Ment Health Syst.

[43] Matcham F, Leightley D, Siddi S (2022). Remote Assessment of Disease and Relapse in Major Depressive Disorder (RADAR-MDD): recruitment, retention, and data availability in a longitudinal remote measurement study. BMC Psychiatry.

[44] Faurholt-Jepsen M, Frost M, Ritz C (2015). Daily electronic self-monitoring in bipolar disorder using smartphones - the MONARCA I trial: a randomized, placebo-controlled, single-blind, parallel group trial. Psychol Med.

[45] Bonilla-Escribano P, Ramírez D, Baca-García E, Courtet P, Artés-Rodríguez A, López-Castromán J (2023). Multidimensional variability in ecological assessments predicts two clusters of suicidal patients. Sci Rep.

[46] Meyerhoff J, Liu T, Kording KP (2021). Evaluation of changes in depression, anxiety, and social anxiety using smartphone sensor features: longitudinal cohort study. J Med Internet Res.

[47] Faurholt-Jepsen M, Frost M, Christensen EM, Bardram JE, Vinberg M, Kessing LV (2020). The effect of smartphone-based monitoring on illness activity in bipolar disorder: the MONARCA II randomized controlled single-blinded trial. Psychol Med.

[48] Lauder S, Chester A, Castle D (2015). A randomized head to head trial of MoodSwings.net.au: an internet based self-help program for bipolar disorder. J Affect Disord.

[49] Goulding EH, Dopke CA, Rossom R, Jonathan G, Mohr D, Kwasny MJ (2023). Effects of a smartphone-based self-management intervention for individuals With bipolar disorder on relapse, symptom burden, and quality of life: a randomized clinical trial. JAMA Psychiatry.

[50] Bilderbeck AC, Atkinson LZ, McMahon HC (2016). Psychoeducation and online mood tracking for patients with bipolar disorder: a randomised controlled trial. J Affect Disord.

[51] White KM, Carr E, Leightley D (2024). Engagement with a remote symptom-tracking platform among participants with major depressive disorder: randomized controlled trial. JMIR Mhealth Uhealth.

[52] Tønning ML, Faurholt-Jepsen M, Frost M (2021). The effect of smartphone-based monitoring and treatment on the rate and duration of psychiatric readmission in patients with unipolar depressive disorder: the RADMIS randomized controlled trial. J Affect Disord.

[53] Aguilera A, Bruehlman-Senecal E, Demasi O, Avila P (2017). Automated text messaging as an adjunct to cognitive behavioral therapy for depression: a clinical trial. J Med Internet Res.

[54] Hunkeler EM, Hargreaves WA, Fireman B (2012). A web-delivered care management and patient self-management program for recurrent depression: a randomized trial. Psychiatr Serv.

[55] Aikens JE, Trivedi R, Heapy A, Pfeiffer PN, Piette JD (2015). Potential impact of incorporating a patient-selected support person into mHealth for depression. J Gen Intern Med.

[56] Helmich MA, Smit AC, Bringmann LF (2023). Detecting impending symptom transitions using early-warning signals in individuals receiving treatment for depression. Clin Psychol Sci.

[57] Dominiak M, Kaczmarek-Majer K, Antosik-Wójcińska AZ (2022). Behavioral and self-reported data collected from smartphones for the assessment of depressive and manic symptoms in patients with bipolar disorder: prospective observational study. J Med Internet Res.

[58] Anýž J, Bakštein E, Dally A (2021). Validity of the Aktibipo self-rating questionnaire for the digital self-assessment of mood and relapse detection in patients with bipolar disorder: instrument validation study. JMIR Ment Health.

[59] Schneider J, Bakštein E, Kolenič M (2022). Motor activity patterns can distinguish between interepisode bipolar disorder patients and healthy controls. CNS Spectr.

[60] Benedyk A, Moldavski A, Reichert M (2023). Initial response to the COVID-19 pandemic on real-life well-being, social contact and roaming behavior in patients with schizophrenia, major depression and healthy controls: a longitudinal ecological momentary assessment study. Eur Neuropsychopharmacol.

[61] Emden D, Goltermann J, Dannlowski U, Hahn T, Opel N (2021). Technical feasibility and adherence of the Remote Monitoring Application in Psychiatry (ReMAP) for the assessment of affective symptoms. J Affect Disord.

[62] Lieberman DZ, Kelly TF, Douglas L, Goodwin FK (2010). A randomized comparison of online and paper mood charts for people with bipolar disorder. J Affect Disord.

[63] Smit AC, Snippe E, Bringmann LF, Hoenders HJR, Wichers M (2023). Transitions in depression: if, how, and when depressive symptoms return during and after discontinuing antidepressants. Qual Life Res.

[64] Aikens JE, Valenstein M, Plegue MA (2022). Technology-facilitated depression self-management linked with lay supporters and primary care clinics: randomized controlled trial in a low-income sample. Telemed J E Health.

[65] Arean PA, Hallgren KA, Jordan JT (2016). The use and effectiveness of mobile apps for depression: results from a fully remote clinical trial. J Med Internet Res.

[66] Perez Arribas I, Goodwin GM, Geddes JR, Lyons T, Saunders KEA (2018). A signature-based machine learning model for distinguishing bipolar disorder and borderline personality disorder. Transl Psychiatry.

[67] Bauer M, Glenn T, Alda M (2023). Longitudinal digital mood charting in bipolar disorder: experiences with ChronoRecord over 20 years. Pharmacopsychiatry.

[68] Bos FM, Schreuder MJ, George SV (2022). Anticipating manic and depressive transitions in patients with bipolar disorder using early warning signals. Int J Bipolar Disord.

[69] Carpenter L, Hindley L, Gonsalves M, Schatten H, Brown J, Tirrell E (2021). App-based ecological momentary assessment and symptom-adaptive scheduling of TMS maintenance treatments to prevent depressive episode recurrence. Brain Stimul.

[70] Castle D, White C, Chamberlain J (2010). Group-based psychosocial intervention for bipolar disorder: randomised controlled trial. Br J Psychiatry.

[71] Dai R (2022). Smart sensing and clinical predictions with wearables: from physiological signals to mental health. McKelvey School of Engineering Theses & Dissertations.

[72] Denicoff KD, Ali SO, Sollinger AB, Smith-Jackson EE, Leverich GS, Post RM (2002). Utility of the daily prospective National Institute of Mental Health Life-Chart Method (NIMH-LCM-p) ratings in clinical trials of bipolar disorder. Depress Anxiety.

[73] Depp CA, Kim DH, de Dios LV, Wang V, Ceglowski J (2012). A pilot study of mood ratings captured by mobile phone versus paper-and-pencil mood charts in bipolar disorder. J Dual Diagn.

[74] Ebner-Priemer UW, Mühlbauer E, Neubauer AB (2020). Digital phenotyping: towards replicable findings with comprehensive assessments and integrative models in bipolar disorders. Int J Bipolar Disord.

[75] Faurholt-Jepsen M, Lindbjerg Tønning M, Fros M (2021). Reducing the rate of psychiatric re-admissions in bipolar disorder using smartphones-the RADMIS trial. Acta Psychiatr Scand.

[76] Frank E, Wallace ML, Matthews MJ (2022). Personalized digital intervention for depression based on social rhythm principles adds significantly to outpatient treatment. Front Digit Health.

[77] Funkhouser CJ, Trivedi E, Li LY (2024). Detecting adolescent depression through passive monitoring of linguistic markers in smartphone communication. J Child Psychol Psychiatry.

[78] Gliddon E, Cosgrove V, Berk L (2019). A randomized controlled trial of MoodSwings 2.0: an internet-based self-management program for bipolar disorder. Bipolar Disord.

[79] Goldberg JF, Bowden CL, Calabrese JR (2008). Six-month prospective life charting of mood symptoms with lamotrigine monotherapy versus placebo in rapid cycling bipolar disorder. Biol Psychiatry.

[80] Klein JP, Berger T, Schröder J (2016). Effects of a psychological internet intervention in the treatment of mild to moderate depressive symptoms: results of the EVIDENT study, a randomized controlled trial. Psychother Psychosom.

[81] Kline EA, Lekkas D, Bryan A (2024). The role of borderline personality disorder traits in predicting longitudinal variability of major depressive symptoms among a sample of depressed adults. J Affect Disord.

[82] Langosch JM, Drieling T, Biedermann NC (2008). Efficacy of quetiapine monotherapy in rapid-cycling bipolar disorder in comparison with sodium valproate. J Clin Psychopharmacol.

[83] Lee HJ, Cho CH, Lee T (2023). Prediction of impending mood episode recurrence using real-time digital phenotypes in major depression and bipolar disorders in South Korea: a prospective nationwide cohort study. Psychol Med.

[84] Lewis KJS, Tilling K, Gordon-Smith K (2023). The dynamic interplay between sleep and mood: an intensive longitudinal study of individuals with bipolar disorder. Psychol Med.

[85] McKnight RF, Bilderbeck AC, Miklowitz DJ, Hinds C, Goodwin GM, Geddes JR (2017). Longitudinal mood monitoring in bipolar disorder: course of illness as revealed through a short messaging service. J Affect Disord.

[86] McIntyre RS, Lee Y, Rong C (2021). Ecological momentary assessment of depressive symptoms using the mind.me application: convergence with the Patient Health Questionnaire-9 (PHQ-9). J Psychiatr Res.

[87] Ortiz A, Park Y, Gonzalez-Torres C (2023). Predictors of adherence to electronic self-monitoring in patients with bipolar disorder: a contactless study using growth mixture models. Int J Bipolar Disord.

[88] Petzold J, Mayer-Pelinski R, Pilhatsch M (2019). Short group psychoeducation followed by daily electronic self-monitoring in the long-term treatment of bipolar disorders: a multicenter, rater-blind, randomized controlled trial. Int J Bipolar Disord.

[89] Pratap A, Atkins DC, Renn BN (2019). The accuracy of passive phone sensors in predicting daily mood. Depress Anxiety.

[90] Stanislaus S, Faurholt-Jepsen M, Vinberg M (2020). Mood instability in patients with newly diagnosed bipolar disorder, unaffected relatives, and healthy control individuals measured daily using smartphones. J Affect Disord.

[91] Tseng YC, Lin ECL, Wu CH, Huang HL, Chen PS (2022). Associations among smartphone app-based measurements of mood, sleep and activity in bipolar disorder. Psychiatry Res.

[92] Turvey C, Fuhrmeister L, Klein D (2023). Secure messaging intervention in patients starting new antidepressant to promote adherence: pilot randomized controlled trial. JMIR Form Res.

[93] Vachon H, Bourbousson M, Deschamps T (2016). Repeated self-evaluations may involve familiarization: an exploratory study related to ecological momentary assessment designs in patients with major depressive disorder. Psychiatry Res.

[94] van den Berg KC, Hendrickson AT, Hales SA, Voncken M, Keijsers GPJ (2023). Comparing the effectiveness of imagery focussed cognitive therapy to group psychoeducation for patients with bipolar disorder: a randomised trial. J Affect Disord.

[95] Webb CA, Murray L, O Tierney A, Forbes EE (2023). Reward-related predictors of symptom change in behavioral activation therapy for anhedonic adolescents: a multimodal approach. Neuropsychopharmacology.

[96] van Genugten CR, Schuurmans J, Hoogendoorn AW (2021). Examining the theoretical framework of behavioral activation for major depressive disorder: smartphone-based ecological momentary assessment study. JMIR Ment Health.

[97] Williams MT, Lewthwaite H, Fraysse F, Gajewska A, Ignatavicius J, Ferrar K (2021). Compliance with mobile ecological momentary assessment of self-reported health-related behaviors and psychological constructs in adults: systematic review and meta-analysis. J Med Internet Res.

[98] Jones A, Remmerswaal D, Verveer I (2019). Compliance with ecological momentary assessment protocols in substance users: a meta-analysis. Addiction.

[99] Seidman AJ, George CJ, Kovacs M (2022). Ecological momentary assessment of affect in depression-prone and control samples: survey compliance and affective yield. J Affect Disord.

[100] Stone AA, Shiffman S (2002). Capturing momentary, self-report data: a proposal for reporting guidelines. Ann Behav Med.

[101] Graham JW (2009). Missing data analysis: making it work in the real world. Annu Rev Psychol.

[102] Jabir AI, Lin X, Martinengo L, Sharp G, Theng YL, Tudor Car L (2024). Attrition in conversational agent-delivered mental health interventions: systematic review and meta-analysis. J Med Internet Res.

[103] Moon E, Chang JS, Kim MY (2012). Dropout rate and associated factors in patients with bipolar disorders. J Affect Disord.

[104] Torous J, Lipschitz J, Ng M, Firth J (2020). Dropout rates in clinical trials of smartphone apps for depressive symptoms: a systematic review and meta-analysis. J Affect Disord.

[105] Astill Wright L, Moore M, Reeves S, Perez Vallejos E, Morriss R (2025). Improving the utility, safety, and ethical use of a passive mood-tracking app for people with bipolar disorder using coproduction: qualitative focus group study. JMIR Form Res.

[106] Gelinas L, Largent EA, Cohen IG, Kornetsky S, Bierer BE, Fernandez Lynch H (2018). A framework for ethical payment to research participants. N Engl J Med.

[107] Moher D, Hopewell S, Schulz KF (2010). CONSORT 2010 explanation and elaboration: updated guidelines for reporting parallel group randomised trials. BMJ.

[108] Reeves BC, Gaus W (2004). Guidelines for reporting non-randomised studies. Complement Med Res.

[109] Astill Wright L, Majid M, Shajan G (2025). The user experience of ambulatory assessment and mood monitoring in depression: a systematic review and meta-synthesis. NPJ Digit Med.

