# Supplementary material for: Dropout, Attrition, Adherence, and Compliance in Mood Monitoring and Ambulatory Assessment Studies for Depression and Bipolar Disorder: Systematic Review and Meta-Analysis
Source: JMIR Ment Health. 2026 Jan 12;13:e83765. doi: 10.2196/83765 (PMC12795494; doi:10.2196/83765)
Supplement: Multimedia Appendix 1 [file mental-v13-e83765-s001.docx]

Search strategy

Search performed 3/3/23. The search strategy was trialled on one database first and then refined subsequently. The search results were uploaded to Rayyan (48). Search terms were determined based on discussion between researchers, previous reviews and consultation with specialist librarians. The search was performed from inception to 3/3/23. The search was updated on 28/10/24.

**Number of abstracts original search 3/3/23:**

Medline: 2984

Embase: 4827

PsychINFO: 3346

SCOPUS: 2321

IEE Xplore: 615

Proquest dissertations and theses global: 2697

Proquest SciTech Collection: 3489

Total: 20,279

Full text review: 565

**Number of abstracts of updated search 3/3/24 – 28/10/24:**

Medline: 494

Embase: 920

PsychINFO: 364

SCOPUS: 1088

IEE Xplore: 99

Proquest dissertations and theses global: 0

Proquest SciTech Collection: 469

Total prior to deduplication: 3236

Auto-deduplicated: 1119

Total: 2117

Full text review: 193

**Published literature:**

| **OVID Medline** | |
| --- | --- |
| 1 | exp bipolar disorder/ OR exp depression OR exp mania/ |
| 2 | (((bipolar or bi polar) adj5 (disorder$ or depress$)) or ((cyclothymi$ or rapid or ultradian) adj5 cycl$) or hypomani$ or mania$ or manic$ or mixed episode$ or rcbd).mp |
| 3 | ('Depressive Disorder' OR 'Depression' OR 'dysthymi*' OR 'affective disorder' OR 'affective disorders' OR 'mood disorder' OR 'mood disorders' OR 'depression*' OR 'depressive*' OR 'dysthymic disorder').mp |
| 4 | 1 OR 2 OR 3 |
| 5 | ('self monitor*' or 'self assess*' or 'self manag*' or 'self record*' or 'self surveillance' or 'patient* monitor*' or 'measurement technolog*' or 'telemonitor*' or 'remote monitor*' or 'passive monitor*' or 'active monitor*' or 'mood track*' or 'mood monitor*' or 'experience sampl*' or 'ecological momentary assessment').mp |
| 6 | 4 adj10 5 |

<https://ovidsp.ovid.com/ovidweb.cgi?T=JS&NEWS=N&PAGE=main&SHAREDSEARCHID=10Q4IjupCc3HoHvVXInK959r2tcDy9vTlJlQsifUVfSVSyNrczwzGqVCqql3svtpo>

| **OVID EMBASE** | |
| --- | --- |
| 1 | bipolar disorder/ or bipolar depression/ or bipolar I disorder/ |
| 2 | depression assessment/ or treatment resistant depression/ or minor depression/ or chronic depression/ or postnatal depression/ or atypical depression/ or antenatal depression/ or adolescent depression/ or "mixed mania and depression"/ or post-stroke depression/ or endogenous depression/ or major depression/ or recurrent brief depression/ or depression/ or bipolar depression/ or perinatal depression/ or agitated depression/ or organic depression/ |
| 3 | "mixed mania and depression"/ or mania/ or bipolar mania/ |
| 4 | (((bipolar or bi polar) adj5 (disorder$ or depress$)) or ((cyclothymi$ or rapid or ultradian) adj5 cycl$) or hypomani$ or mania$ or manic$ or mixed episode$ or rcbd).mp. |
| 5 | ('Depressive Disorder' or 'Depression' or 'dysthymi*' or 'affective disorder' or 'affective disorders' or 'mood disorder' or 'mood disorders' or 'depression*' or 'depressive*' or 'dysthymic disorder').mp. |
| 6 | 1 OR 2 OR 3 OR 4 OR 5 |
| 7 | ('self monitor*' or 'self assess*' or 'self manag*' or 'self record*' or 'self surveillance' or 'patient* monitor*' or 'measurement technolog*' or 'telemonitor*' or 'remote monitor*' or 'passive monitor*' or 'active monitor*' or 'mood track*' or 'mood monitor*' or 'experience sampl*' or 'ecological momentary assessment').mp. |
| 8 | 6 adj10 7 |

https://ovidsp.ovid.com/ovidweb.cgi?T=JS&NEWS=N&PAGE=main&SHAREDSEARCHID=2Ofrc9VijRp6L40USOJFeEn3I1bHOmCW8O1Hzfz13xklneo3jW1767QyyDQMHnVDj

| **OVID PsychINFO** | |
| --- | --- |
| 1 | Bipolar Disorder/ or Bipolar II Disorder/ or Bipolar I Disorder/ or Mania/ |
| 2 | Major Depression/ or Endogenous Depression/ or Postpartum Depression/ or Recurrent Depression/ or "Depression (Emotion)"/ or Reactive Depression/ or Late Life Depression/ or Atypical Depression/ or Treatment Resistant Depression/ or "Long-term Depression (Neuronal)".mp. |
| 3 | (((bipolar or bi polar) adj5 (disorder$ or depress$)) or ((cyclothymi$ or rapid or ultradian) adj5 cycl$) or hypomani$ or mania$ or manic$ or mixed episode$ or rcbd).mp |
| 4 | 'Depressive Disorder' OR 'Depression' OR 'dysthymi*' OR 'affective disorder' OR 'affective disorders' OR 'mood disorder' OR 'mood disorders' OR 'depression*' OR 'depressive*' OR 'dysthymic disorder' |
| 5 | 1 OR 2 OR 3 OR 4 |
| 6 | ('self monitor*' or 'self assess*' or 'self manag*' or 'self record*' or 'self surveillance' or 'patient* monitor*' or 'measurement technolog*' or 'telemonitor*' or 'remote monitor*' or 'passive monitor*' or 'active monitor*' or 'mood track*' or 'mood monitor*' or 'experience sampl*' or 'ecological momentary assessment') |
| 7 | 5 adj10 6 |

https://ovidsp.ovid.com/ovidweb.cgi?T=JS&NEWS=N&PAGE=main&SHAREDSEARCHID=7WPhKe8RR9Athylx2jCCPdAkgbQlgcrdVpxl6NGPhskh73E8wr3X16vfACAP9Q54Y

**SCOPUS:**

TITLE-ABS-KEY({self monitor*} OR {self-monitor*} OR {self-assess*} OR {self manag*} OR {self-manag*} OR {self record*} OR {self-record*} OR {self surveillance} OR {self-surveillance} OR {patient* monitor*} OR {patient*-monitor*} OR {measurement technolog*} OR {measurement-technolog*} OR {telemonitor*} OR {remote monitor*} OR {remote-monitor*} OR {passive monitor*} OR {passive-monitor*} OR {active monitor*} OR {active-monitor*} OR {mood track*} OR {mood-track*} OR {mood monitor*} OR {mood-monitor*} OR {experience sampl*} OR {ecological momentary assessment}) W/10 ({Bipolar disorder*} OR {Manic depress*} OR {Manic-depress*} OR {Bipolar affective psychos*} OR {Bipolar depress*} OR {Manic disorder*} OR (49) OR {depressive disorder*} OR {major depressive disorder*} OR (49) OR {affective disorder*} OR {mood disorder*})

**IEE XPLORE:**

('self monitor' OR 'self monitoring' OR 'self assess' OR 'self assessment' OR 'self manage' OR 'self management' OR 'self record' OR 'self recording' OR 'self surveillance' OR 'patient monitor' OR 'patient monitoring' OR 'measurement technology' OR 'telemonitor' OR 'telemonitoring' OR 'remote monitor' OR 'remote monitoring' OR 'passive monitor' OR 'passive monitoring' OR 'active monitor*' OR 'mood track*' OR 'mood monitor*' OR 'experience sample' OR 'experience sampling' OR 'ecological momentary assessment') NEAR/10 ('Bipolar*' OR 'Manic disorder*' OR 'depressi*' OR 'affective disorder*' OR 'mood disorder*')

**Google scholar search:** An additional search of the first 15 pages of Google Scholar was conducted (search terms: ‘mood track’, ‘ecological momentary assessment’, ‘monitoring’, ‘remote monitoring’, ‘active monitor’, ‘passive monitor’, ‘experience sample’, ‘experience sampling’)

Finally, subject experts were approached to identify additional articles.

**Grey Literature:**

**ProQuest Dissertations & Theses Global:**

(("self monitor" OR "self monitoring" OR "self monitors") OR ("self assess" OR "self assessed" OR "self assessment") OR ("self manage" OR "self managed" OR "self managing") OR ("self record" OR "self recorded" OR "self recording") OR “self surveillance” OR “patient* monitor*” OR ("measurement technologies" OR "measurement technology") OR “telemonitor*” OR ("remote monitoring") OR ("passive monitoring") OR ("active monitoring") OR “mood track*” OR “mood monitor*” OR ("experience sampling") OR “ecological momentary assessment”) NEAR/10 (("bipolar disorder" OR "bipolar disorders") OR ("manic depression" OR "manic depressive") OR “Bipolar affective psychos*” OR ("bipolar depression") OR “Manic disorder*” OR “depressi*” OR ("depressive disorder") OR “major depressive disorder*” OR “depression” OR ("affective disorder" OR "affective disorders") OR ("mood disorder" OR "mood disorders"))

<http://abc.cardiff.ac.uk/login?url=https://www.proquest.com/search/2332884?accountid=9883>

<https://www.proquest.com/pqdtglobal>

**ProQuest SciTech Premium Collection:**

(("self monitor" OR "self monitoring" OR "self monitors") OR ("self assess" OR "self assessed" OR "self assessment") OR ("self manage" OR "self managed" OR "self managing") OR ("self record" OR "self recorded" OR "self recording") OR “self surveillance” OR “patient* monitor*” OR ("measurement technologies" OR "measurement technology") OR “telemonitor*” OR ("remote monitoring") OR ("passive monitoring") OR ("active monitoring") OR “mood track*” OR “mood monitor*” OR ("experience sampling") OR “ecological momentary assessment”) NEAR/10 (("bipolar disorder" OR "bipolar disorders") OR ("manic depression" OR "manic depressive") OR “Bipolar affective psychos*” OR ("bipolar depression") OR “Manic disorder*” OR “depressi*” OR ("depressive disorder") OR “major depressive disorder*” OR “depression” OR ("affective disorder" OR "affective disorders") OR ("mood disorder" OR "mood disorders"))

http://nottingham.idm.oclc.org/login?url=https://www.proquest.com/search/2332894?accountid=8018

<https://www.proquest.com/scitechpremium/>

**Google incognito mode – first 200 results:**

No new papers identified

**Systematic Reviews reference checked:**

These are cited in the main paper
